# Editorial: Exploration of major depressive disorder among children and adolescents: from pathogenesis to intervention

**DOI:** 10.3389/fpsyt.2023.1350201

**Published:** 2024-01-08

**Authors:** Ling Zhang, Huanzhong Liu, Yi Zheng

**Affiliations:** ^1^Department of Psychiatry, Chaohu Hospital of Anhui Medical University, Hefei, China; ^2^Department of Psychiatry, Anhui Psychiatric Center, Hefei, China; ^3^Department of Psychiatry, School of Mental Health and Psychological Sciences, Anhui Medical University, Hefei, China; ^4^Beijing Anding Hospital, Capital Medical University, Beijing Key Laboratory of Mental Disorders, Beijing, China

**Keywords:** major depressive disorder, children, adolescents, pathogenesis, intervention

Major depressive disorder (MDD) is mainly characterized by persistent depression, loss of interest or joy in previously enjoyable activities, recurrent thoughts of death, and physical and cognitive symptoms, which is a serious and highly disabling mental disorder that can cause severely long-term psychosocial impairment (1). MDD among children and adolescents is relatively common. The point prevalence of MDD in children and adolescents was 1.3% in China (2). Childhood depression disorder would increase the probability of depression in adults (3). In addition, plant/body disorders were more common in children and adolescents with MDD (4). Suicide is also a serious issue worth paying attention to in MDD. Among children and adolescents, MDD has the highest mortality risk, with a global incidence rate of 1.3% (5). The average attempted suicide rate of children and adolescents with MDD was 6.27% per year (6). Therefore, there is a high unmet need for exploring prevention and intervention mechanisms for MDD among children and adolescents.

As the guest editors for the Research Topic on the “*Exploration of major depressive disorder among children and adolescents: from pathogenesis to intervention*”, we would like to highlight some research articles here. These articles covered the symptom characteristics, neural characteristics, and interventions of MDD among children and adolescents.

Liu et al. reported that Children and adolescents with MDD had a higher prevalence of underweight, obesity, suicidal ideation, and attempted suicide. When it comes to neural characteristics, Zhang et al. demonstrated that changes in gray matter volume were found in the frontotemporal parietal lobe and subcortical brain regions of MDD adolescents, which have been proven to be related to the severity of depression. In terms of suicidal behavior, Ma et al. have found evidence from neuro-electrophysiology suggested that depressed adolescents who participate in self- injury may experience impaired behavior inhibitory control (BIC) when exposed to self-injury cues.

Wang et al. showed that the prescription and treatment costs of adolescent depression patients have been increasing rapidly, which should arouse our attention. Current antidepressants can improve the function of adolescent MDD patients, but can't improve their quality of life (7). Therefore, there is an urgent need for new and effective treatment methods for children and adolescents with MDD.

Repetitive transcranial magnetic stimulation (rTMS) is a relatively new treatment modality that is gaining attention in the treatment of adolescent depression (8). Zheng et al. demonstrated that Low-frequency rTMS could benefit children and adolescents with MDD. Besides that, Zhao et al. also found that LF-rTMS could improve the NSSI performance and abnormal EEG microstate in children and adolescents with depressive disorders. In addition, formulating treatment plans, Zou et al. demonstrated that it was also necessary to consider the combined effects of sleep and exercise on adolescent depression.

In terms of prevention, Xin et al. stated that actively participating in helping others (this psychological quality) was an important way to prevent and eliminate adolescent depression. In the process of helping others, adolescents enrich their inner emotional experience and construct positive psychological quality, which may form immunity to depression.

Based on the findings of this Research Topic, it is necessary to consider the psychological quality of MDD adolescents in the process of prevention. In addition, when formulating intervention measures, medical personnel need to consider both pharmacological and non-pharmacological treatments such as rTMS, and include consideration of the impact of sleep and exercise on MDD adolescents in the development of treatment plans.

## Author contributions

LZ: Writing – original draft. HL: Writing – review & editing. YZ: Writing – review & editing.

## References

[1] Marx W Penninx B Solmi M Furukawa TA Firth J Carvalho AF. Major depressive disorder. Nat Rev Dis Prim. (2023) 9:44. 10.1038/s41572-023-00454-1 37620370

[2] Xu DD Rao WW Cao XL Wen SY Che WI Ng CH. Prevalence of major depressive disorder in children and adolescents in China: a systematic review and meta-analysis. J Affect Disord. (2018) 241:592–8. 10.1016/j.jad.2018.07.083 30172211

[3] Johnson D Dupuis G Piche J Clayborne Z Colman I. Adult mental health outcomes of adolescent depression: a systematic review. Depress Anxiety. (2018) 35:700–16. 10.1002/da.22777 29878410

[4] Rice F Riglin L Lomax T Souter E Potter R Smith DJ. Adolescent and adult differences in major depression symptom profiles. J Affect Disord. (2019) 243:175–81. 10.1016/j.jad.2018.09.015 30243197

[5] Polanczyk GV Salum GA Sugaya LS Caye A Rohde LA. Annual research review: a meta-analysis of the worldwide prevalence of mental disorders in children and adolescents. J Child Psychol Psychiatry. (2015) 56:345–65. 10.1111/jcpp.12381 25649325

[6] Serra G De Crescenzo F Maisto F Galante JR Iannoni ME Trasolini M. Suicidal behavior in juvenile bipolar disorder and major depressive disorder patients: systematic review and meta-analysis. J Affect Disord. (2022) 311:572–81. 10.1016/j.jad.2022.05.063 35588913

[7] Teng T Zhang Z Yin B Guo T Wang X Hu J. Effect of antidepressants on functioning and quality of life outcomes in children and adolescents with major depressive disorder: a systematic review and meta-analysis. Transl Psychiatry. (2022) 12:183. 10.1038/s41398-022-01951-9 35508443 PMC9068747

[8] Majumder P Balan S Gupta V Wadhwa R Perera TD. The safety and efficacy of repetitive transcranial magnetic stimulation in the treatment of major depression among children and adolescents: a systematic review. Cureus. (2021) 13:e14564. 10.7759/cureus.14564 34026380 PMC8133761

